# Core competencies of radiographers working in rural hospitals of KwaZulu-Natal, South Africa

**DOI:** 10.4102/phcfm.v9i1.1389

**Published:** 2017-08-31

**Authors:** Bernard Mung’omba, Annali D.H. Botha

**Affiliations:** 1 Department of Health Studies, University of South Africa, South Africa; 2 KZN Department of Health, Mosvold Hospital, South Africa

## Abstract

**Introduction:**

Rural radiographers require, over and above traditional radiographic expertise, additional competencies which to a certain degree are unique however not limited to rural practice. Previous studies, however, have focused more attention primarily on other rural health professionals such as doctors and nurses leaving a research need in this field. This article focuses on the additional competencies that may be required for rural radiographers.

**Aim:**

To investigate and identify additional core competencies required by radiographers working in rural hospitals of KwaZulu-Natal in order to propose a continuous professional development strategy aimed at rural radiographers.

**Methods:**

An exploratory sequential design was utilised with qualitative (Phase I) and quantitative (Phase II) strands involving seven participants and 109 respondents, respectively. Only radiographers working in rural KwaZulu-Natal hospitals were included in the study. The four major themes and categories identified in Phase I were used to develop data collection instrument for Phase II of the study.

**Results:**

Collectively, the results revealed that there were a number of additional core competencies such as, but not limited to, teamwork, ability to do basic obstetric ultrasound scans, leadership, management and reporting on plain radiographs, all of which are required by rural radiographers. In 2014 when these competencies were checked against a single curriculum, it was found that majority of them were either partially covered or not at all covered.

**Conclusion:**

The study provides additional information on context specific core competencies and, therefore, may act as a catalyst to influence the future of radiographers working in rural areas of South Africa.

## Introduction

Contribution of general diagnostic imaging in the diagnosis and clinical management of patients and in particular rural hospitals of South Africa is enormous.^1^ A rural district hospital, for the purpose of this article, is defined as a hospital very isolated from urban areas. In many rural hospitals, general radiography remains the first choice of imaging technique.^1,2^ Radiographers are needed to provide radiographic services, as clinical support staff, which is an essential component of the rural health care team. These radiographers acquire core clinical and technical skills during training. The training period referred to here is the pre-introduction of the Bachelor of Science degree by majority of universities of technologies. However, there is a variation both in the context and the required skills in rural areas.^3^ To provide quality health care services in line with the needs of the community requires a workforce that is integrated and is able to demonstrate a range of competencies that are defined by the needs of patients within a particular geographical area.^4^ Rural health care settings make certain demands that may not be anticipated by radiographers during the pre-service training. Ultimately, radiographers working in rural areas need to have both extended and expanded (additional) competencies, which are context specific. These competencies and responsibilities are likely to be beyond the statutory responsibilities and competencies required for professional registration.^5^

According to Strasser et al., the policy document of the Department of Health (DoH) which dealt with the shift towards District Health System based on Primary Health Care (PHC) called for retraining and reorienting of all health workers including radiographers.^6^ Radiographers being part of the rural health care team have important roles to play in rural radiographic management services; however, those working in rural areas appear to lack core expanded and extended competencies that are aligned with the rural environment and are essential for effective performance in rural settings. Despite this situation, there is potential in identifying additional core competencies that may allow for increased effectiveness of senior rural radiographers.^7^ For instance, in other health professions, such as nursing, authors have long advocated the importance of changing the paradigm to a stage of continuing competency development as opposed to just demonstrating competency.^8^ Other than the clinical radiographic practice that rural radiographers need, Boulle suggests that rural health practitioners require skills that may not necessarily be part of the traditional scope.^9^ For instance, Smith and Hays noted that the scope of practice for doctors in rural areas may be extended to encompass that of a specialist.^3^ Similarly, required competencies for rural radiographers may overlap between other rural health disciplines. To this effect, re-engineering of the health care professionals to meet service requirements has been identified as one of the eight thematic priorities in the 2012/13-2016/17 Health Resources for Health strategy.^10^

The complexity and diverse nature of rural radiography practice and the multiplicity of rural patient needs raise the requirements on the competency of radiographers in rural diagnostic imaging departments. To this effect, Engel-Hills is of the view that other than the traditional core radiographic competencies, it was important for the radiography curriculum to integrate generic competencies if it was to meet the demands of the modern work environment.^11^ However, these expanded and extended competency requirements required by rural radiographers at the time may not have been included in the training curriculum.

Nevertheless, high-quality diagnostic imaging services that are context specific require radiographers who have up-to-date skills and expertise. Thus, rural radiographers require, over and above traditional radiographic expertise, context specific competencies which are largely unique to rural practice.^7,12^ To date, however, little is known about which additional core competencies were needed by radiographers working in rural areas. Despite this possible gap, the available studies have focused more attention primarily on other rural health professionals such as doctors and nurses.^4,13,14^ While a great deal has been done on other health professionals such as doctors and nurses, there is very little or no literature on the competency, skills and attributes that may be required to be an effective rural radiographer. There is a need to bring to the attention of health care teams and authorities in rural hospitals the context specific competencies that may be needed by radiographers who deliver diagnostic imaging services in rural hospitals of KwaZulu-Natal (KZN). By identifying additional core competencies required by this professional group, it may be possible to advance and enhance their professional needs. Hence, the aim of this mixed methods study was to investigate and identify additional core competencies required by radiographers working in rural district hospitals of KZN in order to propose a continuous professional development (CPD) strategy aimed at rural radiographers. The process and the development of a proposed CPD strategy is dealt with separately in another article.

## Research methods and design

A mixed method approach was utilised for this study where quantitative and qualitative are combined. By deciding to use mixed method, the researcher sought to compensate the weakness of one method with the strength of another.^15^ An exploratory sequential study design was used. The researcher began by exploring the problem in Phase I before building on to the next phase.^16,17^ This is also the view of Onwuegbuzie and Johnson who write that in sequential type of mixed methods research, one type of data provides a basis for the collection of another type of data.^18^

The validity of this study design was considered according to each phase. It means that the researcher considered separately the trustworthiness and transferability of the results of the qualitative phase. While external and internal validity and generalisability were considered with regard to quantitative phase. This view is supported by many authors on mixed methods research who argue for the use of known procedures for each approach namely qualitative and quantitative.^18,19,20^

## Research setting

The study was conducted on all radiographers working in KZN’s 40 Level 1 rural district hospitals.

## The study population and sampling method

At the time of study, a total of 150 radiographers were identified as working in these 40 rural hospitals. A parallel sample relationship was used. This entails that the sample for focus group interview in Phase I and survey respondents in Phase II were different but drawn from the same population of interest.^21^ There were seven participants in the qualitative phase of the study, whereas in the quantitative phase a census sample of 109 respondents from rural hospitals were surveyed using a postal structured questionnaire.

## Data collection and analysis

### Phase I

In Phase I of the study, data were collected from a single focus group interview of rural radiographers using eight questions. For analysis purposes, the interviews were transcribed from voice-recorded format into text form using the services of professional transcribers. The most important feature of qualitative data analysis is the focus on text rather than on numbers.^22^ In order to understand data, the researcher enacted a close reading to familiarise himself with the transcribed text. This was done by searching through the transcribed data looking for statements and quotes which, according to Padgett, are normally emblematic in meaning.^23^ According to the same author, these emblematic statements and quotes can be grouped into themes.^23^

Four major themes and corresponding categories that emerged from qualitative data analysis in Phase I informed the generation and development of the items in the questionnaire for Phase II. This then connects Phase I strand to Phase II strand, a procedure that Creswell and Clark Plano corroborate.^16^

### Phase II

In Phase II of the study, data were obtained from a census sample using a self-administered structured postal questionnaire. The survey questionnaire consisted of 57 items of which six were open-ended. For all items where Likert scale items were used, the researcher used an even number, namely a four-point (1 – strongly disagree to 4 – strongly agree) Likert scale, deliberately skipping the neutral response. This was done in an attempt to reduce respondent’s manipulation. Similarly, Clason and Dormody also notes that some researchers use an even number for response categories by omitting neutral response to avoid manipulation by respondents.^24^ The questionnaire was pre-tested on eight rural radiographers.

The audit instrument was used to help in the analysis of a curriculum. As the analysis of questionnaire for additional core competencies of radiographers working in rural areas was the focus of this study, categories on the curricula audit checklist were appropriate to the profile of the rural diagnostic radiography area and were based on the literature and results of both qualitative and quantitative data analysis.

### Phase II data analysis

In this study, sequential data analysis was used. The qualitative phase preceded the quantitative phase, and the results from the initial phase analysis were used to inform the subsequent phase.^25,26^ The researcher gave equal importance to both qualitative and quantitative phases of the study, though greater emphasis was placed on the Phase II analysis. According to Onwuegbuzie and Combs, placing emphasis on quantitative phase yields quantitative dominant mixed method data analysis.^26^ However, the rationale for combining qualitative and quantitative data analysis was, in the case of this study, complementary. This means that both phases were given equal weight.

Data entry and analysis were done using Microsoft Excel for Windows 2010 and the Epi-Info version 7.1.5 statistical software programme. Descriptive statistics, namely frequencies, percentages and modes as well as inferential statistics – two groups comparison *t*-test and one-way analysis of variance (ANOVA) were used in the analysis. Percentages were rounded off to one decimal point, and this resulted in percentages adding up to more than 100% or less (e.g. 99.9%) in some cases. For questions to which not all respondents responded, the frequencies and percentages were calculated based on the number of responses. This means that missing values were not included but were indicated in the tables particularly those tables presenting Likert items and open-ended questions. The Likert scale data analysis procedure was used as suggested by Boone and Boone.^27^ This means that sums or averages of the responses on a group of Likert-type items were summed up to give an overall score.^28^ This, according to the same authors, allows for Likert Scale to be used as an interval scale. The level of significance used in the data analysis of relationship in this study was 5% (0.05). In this study, Cronbach’s alpha of 0.65 or higher was used to measure the internal consistency for reliability.

### Ethical consideration

Permission to conduct the study was sought and obtained from both Provincial Health Research and Knowledge Management Committee of the KZN Department of Health (HRKM 091/13) and the University of South Africa’s ethics committee (HSHDC/173/2013). In the case of focus group participants, oral consent was obtained before the interviews. For the survey respondents, it was mentioned in the covering letter that acceptance and completion of the questionnaire constituted consent by the respondent.

## Results

### Phase I

Table 1 reflects the demographic profile of the participants of the focus group. The group comprised of participants with varied characteristics within the radiography profession. Their work experience ranged from 4 years to more than 10 years. There were more female participants (57.1%) than male. The four major themes and corresponding categories and subcategories that emerged from focus group discussion were presented to both the participants and the study supervisor for validation. This process resulted in some changes to the four themes that the researcher had identified earlier. The consensus was reached that one theme, namely ‘challenges encountered by radiographers practising in rural KZN’, should be included under major theme 1 as a category. The remaining three themes are reflected in Table 2.

**TABLE 1:** Demographic characteristics of focus group participants (*n* = 7).

Characteristic	Value	Frequency	Percentage
Gender	Male	3	42.9
	Female	4	57.1
Rank	Assist. Director	2	28.6
	Chief	3	42.8
	Production	2	28.6
Work experience in rural district hospital	4 years	1	14.3
	5 years	1	14.3
	7 years	3	42.8
	More than 10 years	2	28.6

**TABLE 2:** Major themes emerging from Phase I data analysis.

Theme	Description
Theme 1	The environment in which rural radiographers practise radiography
Theme 2	Radiography training and rural radiographic practice
Theme 3	Views of rural radiographers on CPD as a lifelong learning concept

The results obtained through focus group interview were important because they showed in-depth views and opinions, in the rural radiographers’ own words. The type of additional competencies, challenges and other aspects of rural radiography as well as the ways in which these aspects influence their practice was highlighted by participants and some of the direct quotes will be reflected in the Phase II results.

### Phase II

The questionnaires were distributed to 135 radiographers in 38 hospitals. A return rate of 80.7% (*n* = 109) was achieved. Female respondents were more than male respondents representing 65.1% (*n* = 71) and 34.9% (*n* = 38) of the sample, respectively. A little more than half of the respondents 55.1% (*n* = 60) were entry level radiographers, while those with a chief radiographer’s post accounted for 32.1% (*n* = 35). Those holding the post of assistant director of radiography accounted for 12.8% (*n* = 14). The results also showed that 2.8% (*n* = 3) of the respondents held a certificate, while the majority of the respondents 78.9% (*n* = 86) had a national diploma, followed by 17.4% (*n* = 19) of the respondents who held a bachelor’s degree. In this census sample of 109 respondents, only 0.9% (*n* = 1) of the respondents held a master’s degree and no one had a doctoral degree.

The reflections made by participants of this study indicated that one’s ability to work well within the multidisciplinary team was an important competency in rural hospitals. This is consistent with the observation of a community service medical officer who noted that the culture of team work and co-dependence of health care workers in rural hospitals is one thing that lacks in the urban context.^29^ Participants of the focus group and the respondents to the survey all agreed that team work in rural hospitals is essential. ‘The bottom line is that we need each other’ said one participant. However, the results also imply that a proportion of respondents had reservations on radiographer collaborations with team members, especially doctors that may be tainted by the lack of respect. Although the majority (61.5%) responded positively to doctors as members of the rural health care team respecting radiographers 38.5% (*n* = 42) reported otherwise.

Communication was identified as an important competency needed in rural areas. The study shows that communication in rural areas becomes even more important and difficult at the same time because of education levels and cultural beliefs among rural patients.

‘We need to communicate to our patients a bit different. … What I mean is that we need to come down to their level of education and be able to explain in a simple language which they can understand.’ (Female, 38 years, chief radiographer 1)

It is evident from the study results that rural radiographers are expected to employ appropriate language, which is suitable to the recipient of the message, in this case Zulu for patients in rural KZN. Similarly, Chris explains that the foundation of good relation in cultural consultancy was the ability of the health care professional to convey sensible messages to the patient.^30^ Results (Table 3) show that respondents scored themselves positively on all three items except item 17 where a modal score of 3 was observed, indicating that respondents (*n* = 43; 39.5%) answered 3 – agreed. On this item, there was also 20.0% (*n* = 24) of the respondents who did not agree with the statement that rural radiographer leaders foster the skills and development of other radiographers. This positive rating by respondents appears to contradict the assertion that leadership is not traditionally associated with radiography and, therefore, represents additional responsibility which according to some authors is beyond those competencies expected at a point of graduation.^31^ Table 4 shows that item 21 received the highest rating score of 4 implying that 50.9% (*n* = 55) of the respondents strongly agreed that they were able to organise workload and patient flow even when there are few radiographers. The other three items namely 22, 24 and 25 each received a rating score of 3, meaning that the respondents agreed with item statements. This evidence suggests that rural radiographers are capable of executing management duties related to radiography. This, however, is in contradiction with Olsen and Neale, who express concern that this type of management and leadership is seldom included as a part of clinical training.^31^ Olsen and Neal’s concern may be tenable when one considers the results of item 23 where the respondents were divided. An equal percentage 31.5% (*n* = 34) confirmed that they either do not agree or agree with the statement. This implies that those who did not agree were not aware of the budgetary control as related to the required resources for the X-ray unit.

**TABLE 3:** Rural radiography and leadership (*n* = 109).

Item no.	Statement	1 *n* (%)	2 *n* (%)	3 *n* (%)	4 *n* (%)	Mode	Total *n* (%)
16	Rural radiographer leaders are accountable for the professional leadership of a radiographic team	2 (1.8)	15 (13.8)	42 (38.5)	50 (45.9)	4	109 (100)
17	Rural radiographer leaders foster the skills development of other radiographers	9 (8.3)	24 (22.0)	43 (39.4)	33 (30.3)	3	109 (100)
18	Rural radiographer leaders are able to suggest applicable solutions to departmental problems	3 (2.8)	12 (11.0)	42 (38.5)	52 (47.7)	4	109 (100)
19	Rural radiographer leaders use critical thinking skills to support clinical decision-making	4 (3.7)	13 (11.9)	42 (38.5)	50 (45.9)	4	109 (100)

Key: 1 = strongly disagree; 2 = disagree; 3 = agree; 4 = strongly agree.

**TABLE 4:** Rural radiography and management (*n* = 108).

Item no.	Statement	1 *n* (%)	2 *n* (%)	3 *n* (%)	4 *n* (%)	Mode	Total *n* (%)
21	I am able to organise workload and patient flow even when there are few radiographers	3 (2.8)	3 (2.8)	47 (43.5)	55 (50.9)	4	108 (100.0)
22	I am able to participate fully in hospital committees where the interest of radiography is discussed	3 (2.8)	22 (20.3)	46 (42.6)	37 (34.3)	3	108 (100.0)
23	I am aware of budgetary control as related to the required resources for the X-ray unit	8 (7.4)	34 (31.5)	34 (31.5)	32 (29.6)	2, 3	108 (100.0)
24	I am aware of the hospital structure and policies including procedures	2 (1.9)	17 (15.7)	56 (51.9)	33 (30.6)	3	108 (100.1)
25	I am able to manage human resources in the X-ray department effectively	7 (6.5)	17 (15.7)	53 (49.1)	31 (28.7)	3	108 (100.0)

Key: 1 = strongly disagree; 2 = disagree; 3 = agree; 4 = strongly agree.

According to the results, 56.9% (*n* = 62) of the respondents agreed that doctors in rural areas seek the opinions of radiographers on plain radiographs. One participant in Phase I of the study said:

‘Doctors come to ask for instance if I have seen any fracture on the film I have just produced. In rural hospitals we end up becoming ‘radiologists’. Doctors always come to us asking what we think about the X-ray.’ (Female, 25 years, Production 2)

Similarly, findings of a study conducted by the professional board of Radiography and Clinical Technologist (RCT) under the Health Professions Council of South Africa showed that 81.6% of the radiographers who took part reported that the referring clinician asks for their opinion on plain X-rays.^32^ When asked whether rural radiographers perform basic ultrasound scans, two of the respondents did not answer while 75.7% (*n* = 85) who did answer the question indicated that rural radiographers were carrying out basic ultrasound scans. Similarly, Thulo noted that because of the shortage of radiologists especially in rural areas, radiographers take up roles which include ultrasound, report writing and administration of intravenous contrast.^33^

Data reveal a bi-modal (1, 2) for item 29 (Table 5). It is observed that 32.1% (*n* = 35) of the respondents answered ‘strongly disagreed’ with item 29 statement while 31.2% (*n* = 34) indicated disagree. This entails that out of 109 respondents 69 were of the opinion that the radiography curriculum does not provide radiography students with exposure to rural settings during training. For item 30, 34.9% (*n* = 38) of the respondents agreed with the statement while 29.4% (*n* = 32) did not agree with the statement. This concurs with Reid and Cakwe who noted that as students’ learning is driven by assessment, it is important that knowledge, skills and attitude relevant to rural practices are included in the curriculum.^13^ Of the 109 respondents, 58.6% (*n* = 61) were of the opinion that the existing scope of practice for radiography was limiting rural radiographers on the role they need to fulfil. Results from both phases of the study revealed that radiographers working in rural areas were faced with a wide range of challenges, some of which are more prevalent in rural radiography practice. For instance, 78.9% (*n* = 86) of the respondents reported that doctors in rural areas normally send patients for unnecessary X-ray examinations. Other challenges that emerged from the study included unnecessary demand by rural patients to be X-rayed, as well as the use of old equipment. Results show that 72.5% (*n* = 79) of respondents agreed that rural radiographic practice is characterised by patient demand for X-ray examination even when the examination is not indicated. However, 27.5% (*n* = 30) of respondents disagreed. These results are comparable to those obtained by Mung’omba and Botha in a study on factors influencing patients demand for X-ray examination in rural areas of KZN.^34^ Results from both phases of this study showed that participants and a 38.5% proportion of respondents cited a lack of respect and appreciation by fellow team members, with reference to the doctors in particular. Lack of respect for these selected groups maybe the underlying cause of the ‘not so good’ relationship between rural doctors and radiographers. The relationship between rural radiographers and the doctors referred to in this study is characterised by resentment among these radiographers. This is reflected in one participant’s comment:

‘Rural radiographers should be taken as professionals not the way it is happening now. We need respect and to be consulted anytime they need information be it radiography or ultrasound.’ (Male, 28 years, chief radiographer)

**TABLE 5:** Radiography training (*n* = 109).

Item no	Statement	1 *n* (%)	2 *n* (%)	3 *n* (%)	4 *n* (%)	Mode	Total *n* (%)
29	The radiography curriculum provides for exposure of radiography students to rural setup during training	35 (32.1)	34 (31.2)	29 (26.6)	11 (10.1)	1, 2	109 (100.0)
30	The training equipped me to be able to adapt to the demands of rural radiography practice	17 (15.6)	32 (29.4)	38 (34.9)	22 (20.2)	3	109 (100.1)
31	A radiographer is able to maintain a film processor including cleaning and changing chemicals	3 (2.6)	17 (15.6)	48 (44.0)	41 (37.6)	4	109 (100.0)
32	My training enables me to formulate innovative solutions to rural radiographic challenges	6 (5.5)	25 (22.9)	49 (45.0)	29 (26.6)	3	109 (100.0)
33	Rural radiographers need short courses to supplement their current skills	7 (6.4)	12 (11.0)	37 (33.9)	53 (48.6)	4	109 (99.9)

Key: 1 = strongly disagree; 2 = disagree; 3 = agree; 4 = strongly agree.

It is generally apparent from the study results that these radiographers struggle to adjust and adapt to rural challenges. The failure to adapt and adjust may be linked to lack of support and absence of radiologists, which have been identified as challenges in both phases of the study. Data from this study show that 83.5% (*n* = 91) reported that absence of specialists such as radiologists affects the work of radiographers.

## Discussion

The results from both phases of this study imply that radiographers working in rural areas were confronted with a variety of challenges and responsibilities that require a broad range of skills and competencies. It appears that rural radiographers, even without extra competency development, undertake a broad range of simple and complex duties during the course of their practice. The study reflects both clinical and non-clinical competencies that radiographers may be expected to develop while working in rural areas many of which may not have been anticipated at a point of graduation, but are essential for rural practice. For instance, leadership might be one of the competencies which traditionally may not be associated with radiography but it is unique to rural radiographic practice and represents an expanded responsibility and technical competency, which according to Hardy and Snaith, is beyond those expected at the point of registration.^5^

It is essential to reinforce management skills and integrating them into practice. The study shows that respondents were equally divided on the issue of budgetary control. It is important that rural radiographic managers take cognisance that every decision made must ensure equitable delivery of radiographic services. This is necessary because in many instances rural radiography is practised in the context of numerous and significant challenges such as budgetary and resource constraints.

Notwithstanding various opinions regarding the interpretation of conventional X-ray images done by radiographers on a regular basis, it is now confirmed that radiographers give opinions within the context of rural hospitals. Results of this study substantiate why rural radiographers consider interpretation or reading of conventional X-ray images as a desired attribute that they must be skilled in. The ability to better interpret basic skeletal radiographs may enable the rural radiographers to initiate further projections. In the same vein other authors also noted that it is a core competency that will increase the ability of rural radiographers to further provide quality imaging service.^35,36,37^

Despite the ongoing battle of Health Professions Council of South Africa (HPCSA) not allowing diagnostic radiographers to perform such duties, and the need for accreditation of specialised procedures such as contrast injections, reporting on accident and emergency radiographs or basic ultrasound evaluations,^38,39^ reports from the survey show that diagnostic radiographers do perform informal basic ultrasound scans within the rural hospital context. This demonstrates a preference by rural radiographer respondents to being honest in stating that they are doing ultrasound scans illegitimately. This means that the rural radiographer’s professional borders continue to fade and is influenced by factors such as skills shortage and work environment. This, however, points to an existing potential for increased competency expansion for rural radiographers and a better preparation for them to meet both existing and emerging responsibilities. The urgent need for CPD accreditation of rural radiographers performing ultrasound is thus vital. The evidence suggests that the scope of radiography in this particular setting is narrow and may need to be extended. Similarly, the DoH’s HRH strategy for 2012/13-2016/17 proposes that one should consider how the scope of work and job design for mid-level workers could be adjusted through task shifting or sharing.^10^ This is likely to accommodate a broad range of competencies required by rural radiographers. The competencies enshrined in the scope of radiography practice should not be rigid but flexible as different environments in which radiography is practiced may have an influence on radiographer skills development and specialised training, such as ultrasound and X-ray reporting to name a few. The researcher suggests that it may be worthwhile for the scope of radiographic practice in rural areas not to mirror the radiography profile associated with urban practice, but to improve competencies through CPD training activities specific to rural practice. This assertion is supported by Cooke and Couper who also argue that the scope of practice must not impact on rural practice but must be flexible and appropriate for rural areas.^40^

The continuous increase in the demand for extra responsibilities and the blurring boundaries in the duties being undertaken by rural radiographers suggest that their training needs to be responsive to the demands of rural radiographic practice in order to meet the needs of the changing rural health care priorities. Having a multi-skilled and competent rural radiographer will, for instance, use part of his or her hospital position to lead in a number of ways both formal and informal such as self-direction that involves applying knowledge that is supported by evidence. These results may have an implication in that most radiographers working in rural areas may lack the diverse competencies and qualifications that are required to cope with the demands of rural radiography practice. Similarly, participants who took part in a study conducted by Smith also mentioned that there was limitation in the training provided to prepare and support medical practitioners in rural practice.^41^ This again justifies the need for the proposition of a CPD strategy aimed at rural radiographers

## Limitation of the study

The limitations of this study are as follows. Firstly, the design of this study only allowed for auditing of a single radiography curriculum. It must be emphasised that the audit that was done by the researcher on the curriculum was not a curricular quality control audit, but merely done to establish the extent of the areas identified in this study that were either covered or addressed by the single institution or not. Secondly, the study population was limited to one province, which reduces the generalisability of results to the wider population of rural radiographers in South Africa. Thirdly, the competency framework that guided this study was limited in that it only focused on the core competencies of radiographers working in rural areas of KZN. It may require further adjustment such as comparisons to urban environments if it is to be applied nationally. In view of the above-mentioned limitations, it is worthwhile to remind the reader to take into account what the study claims in the text and conclusions made thereof. It must, however, be stated that despite the limitations outlined above, this research is an important research that may be used as a departure point for other studies.

## Recommendations

Below are some of the general recommendations based on the study results:

- Skills development programmes should be made accessible to all diagnostic radiographers working in rural areas.
- Radiography graduates should be guided and be flexible enough to adapt to the ever changing rural health practice and the consequent internal integration. Internal integration will eventually support the successful adoption of external setting.
- Communication and articulation among stakeholders, namely radiography training institutions, rural radiographers and employers, should be enhanced or be introduced if non-existent.
- Radiographers should work towards having representation at district level and at head office. This will afford the radiographers a platform which can be used to air their work requirements and needs.
- Radiography training institutions must acknowledge and support rural radiography CPD programmes that are specifically designed for rural radiographic practice to be able to help enhance competency needs.

## Future research

In terms of further research the following recommendations are made:

- It is recommended that future research may consider a countrywide survey of rural radiographers’ competencies.
- All curricula of the country’s radiography training institutions could be analysed and compared in order to determine specific radiographer competency outcomes that test whether the graduates are able to cope and perform efficiently in rural radiography practice.
- This research may be used as a reference for studies focusing on other rural health professionals other than radiographers.

## Implications for rural radiography in South Africa

This study aimed to identify additional core competencies for rural radiographers of KZN in order to propose a CPD strategy to address much that follows. This research thus advances the understanding of expanded core competencies required to effectively practise rural radiography which may be of importance to both radiography training institutions and rural district hospital managers. The majority of the identified competencies and responsibilities are beyond the statutory responsibilities and competencies required for professional registration. Therefore, those who are involved in the restructuring of radiography in South Africa as a profession, namely training institutions, the DoH and the professional board for radiography and clinical technology under HPCSA, will have to pay attention to the evidence emanating from this study and other similar studies. These institutions should strive towards empowering individual rural radiographers to fulfil their potential

## Conclusion

This study offers reported information to support the suggestion that expanded competencies needed by rural radiographers are not just focused on radiologist-led domains but also on areas that are traditionally the domain of other professionals outside the diagnostic imaging terrain. It is now evident that rural radiographers (particularly those performing ultrasound scans without certification) may not be able to continue practising radiography based solely on the competencies they obtained at the point of graduation, and neither can they rely on the scope of radiography practice which appears to focus on minimum competencies that may hinder the expansion of radiography practice in rural areas. It is hoped that this study provides additional core competencies that appear important for rural radiography and may act as a catalyst to influence the future of radiographers in South Africa, and possibly in comparable environments in the countries of the sub-region.
